# Hyperglycemic Stress Impairs the Stemness Capacity of Kidney Stem Cells in Rats

**DOI:** 10.1371/journal.pone.0139607

**Published:** 2015-10-02

**Authors:** Guang Yang, Yali Jia, Chunlin Li, Qingli Cheng, Wen Yue, Xuetao Pei

**Affiliations:** 1 Department of Geriatric Nephrology, Chinese PLA General Hospital, State Key Laboratory of Kidney Disease, Beijing, China; 2 Stem Cell and Regenerative Medicine Lab, Beijing Institute of Transfusion Medicine, Beijing, China; 3 Department of Geriatric Endocrinology, Chinese PLA General Hospital, Beijing, China; Center for Molecular Biotechnology, ITALY

## Abstract

The incidence of acute kidney injury in patients with diabetes is significantly higher than that of patients without diabetes, and may be associated with the poor stemness capacity of kidney stem cells (KSCs) and limited recovery of injured renal tubules. To investigate the effects of hyperglycemic stress on KSC stemness, KSCs were isolated from the rat renal papilla and analyzed for their self-renewal and differentiation abilities. Our results showed that isolated KSCs expressed the mesenchymal stem cell markers N-cadherin, Nestin, CD133, CD29, CD90, and CD73. Moreover, KSCs co-cultured with hypoxia-injured renal tubular epithelial cell (RTECs) induced the expression of the mature epithelial cell marker CK18, suggesting that the KSCs could differentiate into RTECs in vitro. However, KSC proliferation, differentiation ability and tolerance to hypoxia were decreased in high-glucose cultures. Taken together, these results suggest the high-glucose microenvironment can damage the reparative ability of KSCs. It may result in a decreased of recovery capability of renal tubules from injury.

## Introduction

The incidence of type 2 diabetes is rapidly rising, and affects ~9.7% of the Chinese population [1]. Diabetes is closely associated with kidney diseases because of its resulting effects on blood flow dynamics, oxidative stress, and glucose metabolism in the kidneys [2]; thus, the renal functional reserve capacity is significantly lower in diabetic patients than that of healthy individuals. As such, diabetics are markedly more prone to acute kidney injuries (AKIs) and exhibit retardation of kidney function recovery and a worse prognosis [3,4]. Our recent research has shown that renal tubular interstitial lesions already exist at the early stages of diabetic kidney disease [5]. Since renal tubular interstitial injury in diabetes is closely associated with inferior renal function [6], we speculate that the diminished kidney functional reserve capacity in these patients may result from the due to the impaired recovery of injured renal tubules.

Renal tubular epithelial cells (RTECs) display high regenerative capacities necessary for the quick repair of renal tubules following ischemia- or toxicity-induced AKI. Most studies show that damaged RTECs are replaced by regenerated RTECs that originate from three primary sources: (1) adjacent RTECs phenotypic transfer (mitosis), (2) bone-marrow-derived mesenchymal stem cells (MSCs), or (3) innate kidney stem cells (KSCs). Currently, most researchers recognize that the self-regeneration of RTECs relies on resident kidney stem cells, rather than MSCs [7]. In 2004, Oliver *et al* first reported that KSCs likely reside in the stem cell niche of the renal papilla and provided an early confirmation of their involvement in the recovery of ischemic renal tubular injury. They also observed that KSCs migrated into damaged lesions via chemotaxis, where they subsequently differentiated into RTECs [8,9]. However, because of the technical difficulty of isolating and cultivating the cells from this location, reports regarding KSCs-related biological properties are sparse.

In our previous study, we successfully isolated KSCs from the rat renal papilla [10], and observed their ability to protect and participate in the repair of ischemic/reperfusion-induced renal tubule injury. The protective effects of the KSCs are notably stronger than that of bone-marrow-derived MSCs in rats [11]. In this study, to evaluate the effects of hyperglycemic stress (a combination of high glucose and osmotic stress) on the biological capacity of the KSCs, we isolated KSCs from the rat renal papilla cultured them in high-glucose cell culture medium to mimic the physiological microenvironment of patients with diabetes. Cellular function—with respect to their oxidative stress responses, proliferation, and differentiation into RTECs—was then evaluated.

## Materials and Methods

### Animals, cells, and ethics statement

Four-week-old, specific pathogen-free, male, Sprague Dawley rats (n = 10) were supplied by the experimental animal research unit of Beijing Union Medical College, China Academy of Medical Sciences (License No: SCXK [Jing] 20090007). The NRK-52E rat RTEC line was purchased from the Shanghai Cell Library, Chinese Academy of Sciences. This project was approved by the Animal Care and Use Committee of Chinese PLA General Hospital.

### KSCs isolation and culture

KSCs were isolated from rats euthanized with CO_2_ under isoflurane anesthesia and all efforts were made to alleviate suffering. Both kidneys were excised, the renal capsules stripped, and a longitudinal incision was created along the coronal axis of the kidney under a dissecting microscope to visualize the petal-like papillary structure when the ureter was lifted. The renal papillary tip was removed with microscissors and placed in an Eppendorf tube. The tissue washed with PBS buffer, cut into pieces, and fully digested with collagenase type I (2 mg/mL in PBS buffer, Sigma, USA) for 30 min in a 37°C water bath. The cell suspension was then centrifuged, the supernatant discarded, and the cells cultured with MSC medium (Beijing Jin Ze Xiao Xing Biotechnology Co. Ltd., China) containing 5.6 mM and 6% fetal calf serum (FCS) in a 37°C incubator with 5% CO_2_. MSCs medium was replenished every 2~3 days, with 1:2 passages performed once cells reached 70%~80% confluency. NRK-52E cells were cultured in Dulbecco’s modified Eagle’s medium and Ham’s F-12 nutrient mixture (DMEM/F-12, 3:1) medium (Gibco, USA) with 10% fetal bovine serum (FBS) (Gibco, USA) and passaged 1:5 every three days.

### Immunofluorescence

Passage 3 (P3) KSCs were fixed in 4% paraformaldehyde/PBS buffer in a 24-well plate (Corning Inc., USA) at room temperature, permeabilized with 0.1% Triton X-100 (Beijing Chemical Factory), blocked for 30 min in 10% goat serum (BD, USA), and then incubated with primary antibodies at 4°C overnight. The primary antibodies included mouse anti-E-cadherin polyclonal antibody, rabbit anti-ZO-1 polyclonal antibody, rabbit anti-fibronectin polyclonal antibody (Santa Cruz, USA), mouse anti-N-cadherin polyclonal antibody, mouse anti-α-SMA polyclonal antibody (Sigma, USA), and rabbit anti-Vimentin polyclonal antibody (Cell Signaling Technology, USA). The secondary antibodies—tetramethylrhodamine isothiocyanate (TRITC)-labeled goat anti-rabbit IgG, goat anti-mouse IgG, fluorescein isothiocyanate (FITC)-labeled goat anti-rabbit IgG, or goat anti-mouse IgG (Beijing Zhongshan Golden Bridge Biotechnology Co. Ltd., China)—and 4′-6-diamidino-2-phenylindole (DAPI, BD, USA) were added sequentially. Imaging was performed with an IX70 confocal fluorescence microscope (Olympus, Japan).

### Flow Cytometry

P3 KSCs were suspended in 100 μL PBS buffer pH 7.4 and labeled with allophycocyanin (APC)-conjugated monoclonal rat CD29 antibody (Biolegend, USA), phycoerythrin (PE)-conjugated monoclonal CD90 antibody (BD, USA), or peridinin chlorophyll protein (PerCP)-conjugated monoclonal CD45 antibody against (Biolegend, USA) for 30 min at 4°C. For CD73 immunostaining, KSCs were incubated with 2 μL mouse anti-rat CD73 antibody (BD, USA) at 4°C on a shaker for 30 min, followed by AlexaFluor 647-conjugated goat anti-mouse IgG secondary antibody (Invitrogen, USA). Cells were washed three times in cold PBS buffer and then analyzed on a BD FACSCalibur (BD Biosciences, Canada).

### Identification of Adipogenic and Osteogenic KSC Differentiation

#### Adipogenic differentiation

P3 KSCs were plated at 10^4^ cells per well into 24-well plates and cultured in adipogenic differentiation media (Cyagen, USA) according to the manufacturer’s instructions. Briefly, solution A was added to the cultured KSCs for three days, and then replaced with solution B for one day. This alternating cycle was repeated three times. KSCs were subcultured in solution B for seven days, stained with oil red O, and adipogenic differentiation was then assessed under an inverted microscope (Olympus, Japan).

#### Osteogenic differentiation

KSCs were plated at a density of 5 × 10^3^ cells per well in 24-well plates, allowed to adhere, and then cultured in osteogenic induction medium (Cyagen, USA) with media changes every three days. Two weeks later, KSCs were stained with alizarin red to observe early osteogenic differentiation.

### Identification of Epithelial KSC Differentiation

**Fig 1:**
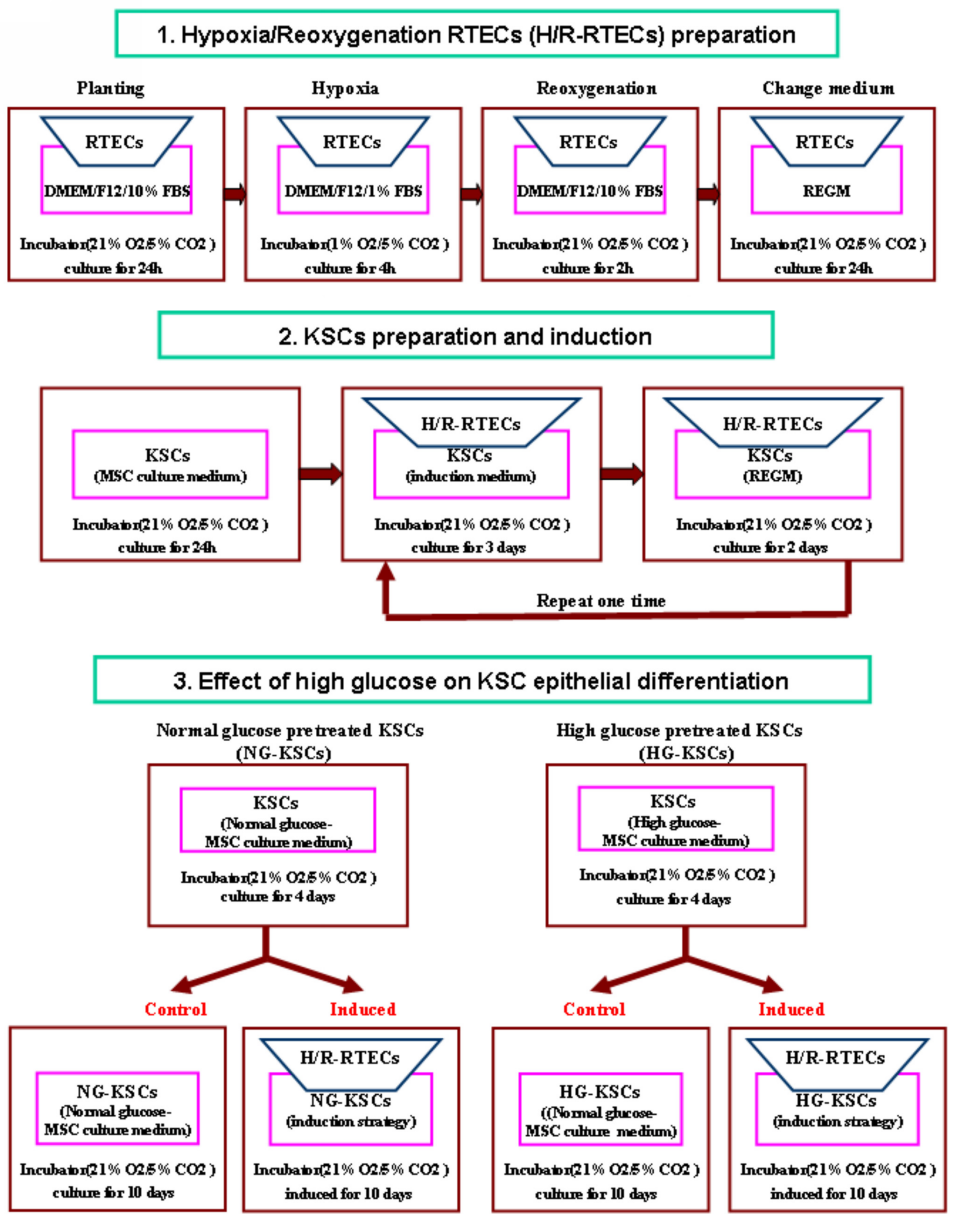
Schematic of the KSC epithelial induction protocol. KSC epithelial differentiation was determined by Transwell co-culture differentiation assay. Hypoxia-injured RTECs and KSCs were prepared and plated in the Transwell inserts and wells, respectively. Cells were co-cultured in induction media for three days, moved to renal epithelial cell growth medium (REGM) for two days, and then changed back to induction medium. This series was repeated twice for a total 10 days. The inserts containing hypoxia-injured RTECs were renewed every three days.

Epithelial KSC differentiation was evaluated by Transwell co-culture differentiation assay (0.4 μm membrane diameter, Corning Inc., USA). First, hypoxia-injured RTECs (2 × 10^3^ RTECs plated in Transwell inserts) were prepared using the following sequential culture protocol: (1) DMEM/F12/10% FBS media in a 21% O_2_/5% CO_2_ atmosphere for 24 h; (2) DMEM/F12/1% FBS in a 1% O_2_/5% CO_2_ atmosphere incubator (Heraeus, Germany) for 4 h; (3) DMEM/F12/10% FCS in a 5% CO_2_ atmosphere for 2 h; (4) renal epithelial cell growth medium (REGM) (Lonza, Switzerland) and 21% O_2_/5% CO_2_ atmosphere for 24 h. Second, the KSCs were plated at 10^4^ cells per well into 24-well plates. Co-cultures were then performed by transferring the inserts containing injured RTECs into the KSCs wells and induction medium, including 10 ng/mL activin A (PeproTech), 10 ng/mL BMP-7 (PeproTech), and 5 μM of retinoic acid (Sigma, USA), was added. Three days later, the culture media was replaced with REGM for two days, and then changed into induction medium again. This sequence was repeated twice for a total 10 days. The inserts containing hypoxia-injured RTECs were renewed every three days. Following the immunofluorescence protocol described above, KSCs were stained with mouse anti-CK18 polyclonal antibody (Abcam, UK), mouse anti-E-cadherin polyclonal antibody, and rabbit anti-ZO-1 polyclonal antibody (Santa Cruz, USA). The induction strategy is shown in Fig 1.

To assess the effect of hyperglycemic stress on KSC epithelial differentiation, cells were cultured in high- or normal-glucose MSC culture medium (30 mM or 5.6 mM D-glucose, respectively) for four days prior to plating at 10^5^ cells per well into the lower compartment of a Transwell plate. Cell adherence was observed after 24 h. Four experimental groups were divided as follows: high-glucose induced, normal-glucose induced, high-glucose control, and normal-glucose control. Induced KSCs were subjected to the culturing procedure detailed above, while control cells were cultured with normal-glucose MSC media for 10 days. The percentage of CK18-positive cells, and E-cadherin and AQP-1 gene expression was determined by flow cytometry and qRT-PCR, respectively.

### Quantitative real-time PCR (qRT-PCR)

Differences in KSC and RTEC gene expression were compared by quantitative real-time PCR (qRT-PCR) using cDNA reverse transcription and qPCR expansion kits (SYBR Green) purchased from TOYOBO (Japan). Total RNA was extracted using TRIzol (Life Technologies) and used to synthesize cDNA, which was used as template in qRT-PCR reactions. Primer sequences were as follows: *Gapdh*: forward, 5′-AGACAGCCGCATCTTCTTGT-3′, reverse, 5′-TTCCCATTCTCAGCCTTGAC-3′; *Nanog*: forward, 5′-GAAGACTAGCAACGGCCTGACT-3′, reverse, 5′-GGTTTCCAGACGCGTTCATC-3′; *Oct4/Pou5f1*: forward, 5′-GGCTGGACACCTGGCTTCAGA-3′, reverse, 5′-TGGTCCGATTCCAGGCCCA-3′; *Sox2*: forward, 5′-CACCATGGCGACCGGCGGCAACCAG-3′, reverse, 5′-TCAAAGCGTGTACTTATCCTTCTTC-3′; *AQP1*: forward, 5′- GGCTTGTCTGTGGCTCTTG -3′, reverse, 5′- ATCATCAGCATCCAGGTCATAC -3′; *E-cadherin*: forward, 5′- AAAGCAGGAAGAAAACACCACTC -3′, reverse, 5′- AAAGGGCACGCTATCAACATTAG -3′. PCR mixtures consisted of 10 μL qPCR mix, 1 μL primer, 1 μL cDNA, and 8 μL nuclease-free water for a total reaction volume of 20 μL. The reaction conditions were as follows: 95°C for 2 min; 45 cycles of 95°C for 15 sec, 58°C for 20 sec, 72°C for 30 sec, and 80°C for 10 sec; 95°C for 10 s. All reactions were performed in triplicate using a Bio-Rad IQ5 system (Bio-Rad, Hercules, CA, USA).

### Western blotting

Differences in KSC and RTEC protein expression were determined by western blotting. For this, cell protein was extracted using RIPA buffer (50 mM Tris–HCl pH 7.5, 150 mM NaCl, 1% NP-40, 0.5% sodium deoxycholate, 0.1% SDS, protease inhibitor cocktail [Set I, Calbiochem, USA]). Extracts were separated by 10% SDS-PAGE and transferred onto polyvinylidene difluoride membranes (PVDF, Bio-Rad). Membranes were blocked with TBST containing 5% skim milk and incubated with primary antibodies overnight at 4°C. After washing with TBST, membranes were then incubated with horseradish peroxidase (HRP)-conjugated secondary antibodies (Beijing Zhongshan Biotechnology, Beijing, China) for 1 h at room temperature. After three additional washes with TBST, reactive bands were visualized by enhanced chemiluminescence (Santa Cruz Biotechnology, Santa Cruz, CA). The antibodies used for western blots were obtained as follows: rabbit polyclonal OCT4 antibody (11263-1-AP, Proteintech), rabbit polyclonal SOX2 antibody (11064-1-AP, Proteintech), and mouse monoclonal β-actin antibody (A1978, Sigma).

### KSC proliferation assays

KSC proliferation monitored using a Cell Counting Kit-8 (CCK-8) (Dojindo, Japan). KSCs were cultured in 96-well plates at 10^3^ cells per well. After the cells adhered, the culture medium was replaced with high-glucose MSC (30 mM D-glucose) or normal-glucose control MSC medium (5.6 mM D-glucose). Media was replenished every three days. At the fixed time point, the medium was replaced with CCK-8 complete culture solution, incubated for 3 h, and then the optical density at 450 nm (OD450) of the culture media was measured and used to calculate growth curves. The total preceding measurement was performed continuously for 7 days. Population doubling times were calculated using the following equation: TD = T × log_2_/ (logN_t_-logN_0_), where TD is the population doubling time, T is the cultivation time, N_0_ is the OD at the start of exponential growth, and N_t_ is the OD value after T hours of exponential growth.

### Hypoxia-induced cell death analysis

KSCs were plated at 2 × 10^4^ cells per well in 6-well plates and cultured for 24 h, at which point the culture medium was replaced with either high-glucose or normal-glucose MSCs medium and the cells cultured for another 96 h. Then, all cells were replated in normal-glucose medium and transferred to a hypoxic incubator with a 1% O_2_ atmosphere for 24 h. Cells were then stained with propidium iodide (PI) to examine cycle progression. For this, harvested cells were resuspended in a single-cell suspension and washed twice with cold PBS buffer, fixed in 70% ethanol at -20°C for 12 h, and washed twice with PBS buffer. Fixed cells were suspended in 100 μL PBS buffer, and 5 μL RNase (10 mg/mL) was added to denature RNA at 37°C for 30 min. Then, 300 μL PI was added to the cell suspension in the dark for 10–15 min. PI staining was detected by flow cytometry and cell cycle progression analyzed with ModFit LT software (Verity Software House, USA). Cell viabilties were calculated using the following equation: Viability (%) = (S + G2/M)/(G0/G1 + S + G2/M) × 100%, where S, M, and G represent the cell cycle phases.

### Statistical analysis

SPSS 13.0 software was used for statistical analysis. Data are expressed as the mean ± standard deviation (SD). Pairwise comparisons were analyzed by group *t*-test. P < 0.05 was considered statistically significant.

## Results

### Morphological characterization of renal papillary cells

**Fig 2:**
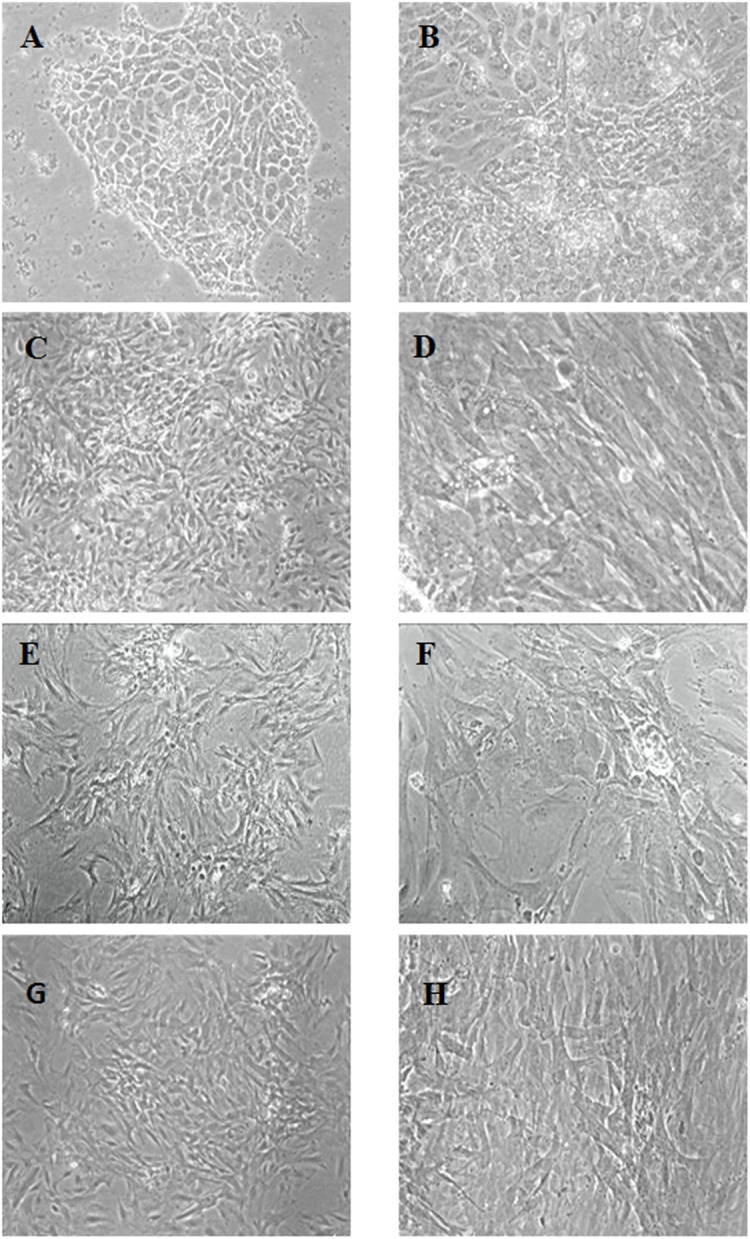
Morphological characterization of renal papillary cells. (A) Primary cell culture on day 3. Cells exhibited colony-like growth and diverse morphology. (B) Primary cell culture on day 5. Epithelioid and fibroblast-like morphologies were observed. (C,D) P2 cells after passage on day 5. (E,F) P3 cells showed a short fusiform or dendritic shape, and epithelioid cells had almost completely disappeared. (G,H) P5 cells exhibited a spindle-shape. (Magnification: C, E, G, ×40; A, B, F, H, ×100; D, ×200).

Renal papillary cell adherence was observed 24 h after isolation. Notably, the cells displayed colony-like growth with diverse morphology after 48 h of culture, and primarily consisted of epithelioid and fibroblast-like cells. After passaging at a 1:2 ratio, P3 subculture cells showed a short fusiform or dendritic shape, and the epithelioid cells had almost completely disappeared. Remaining cells appeared densely fibrous with a large nucleus, and some contained multiple nuclei (Fig 2).

### Phenotypic characterization of renal papillary cells

**Fig 3:**
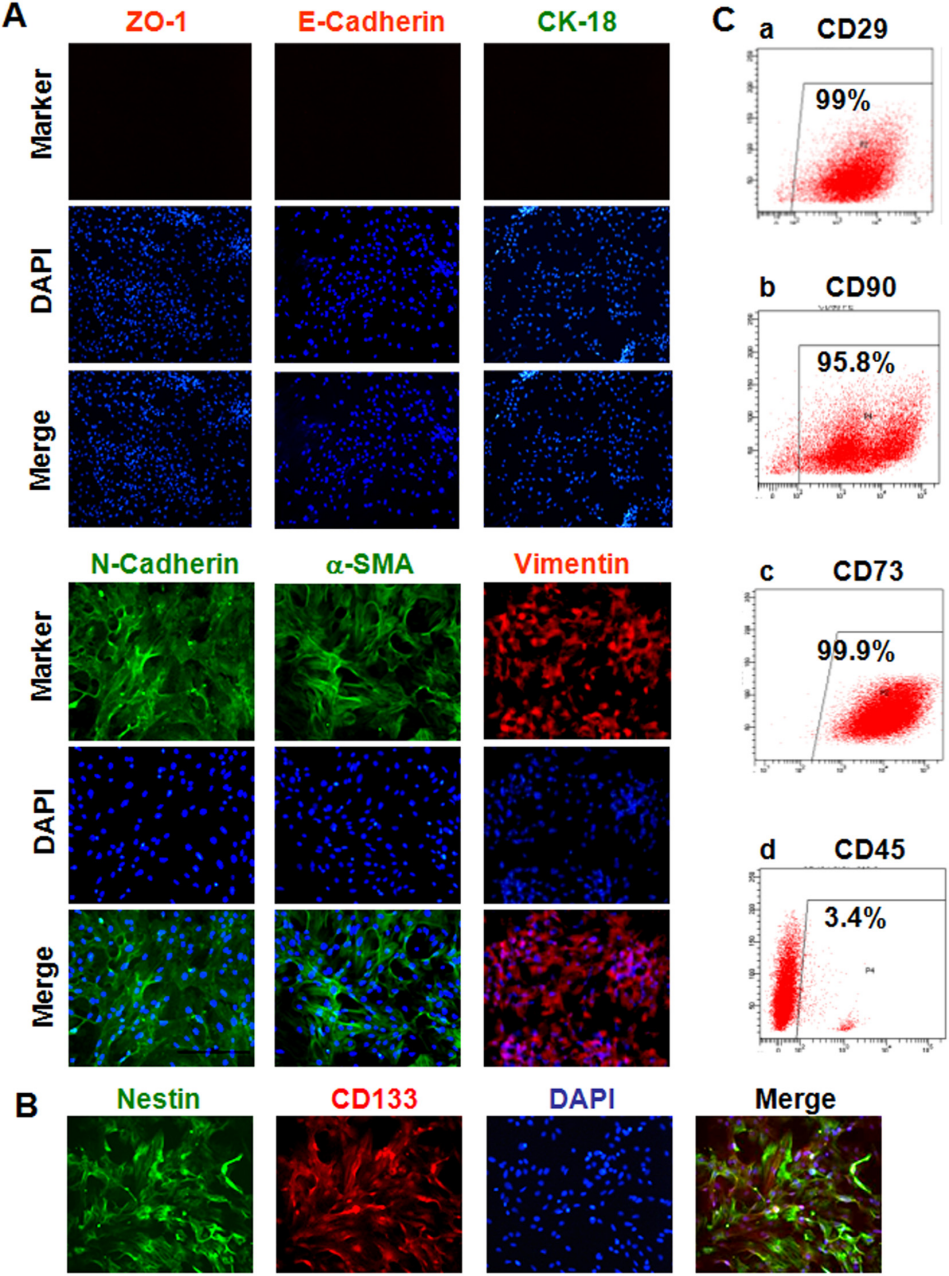
Phenotypic characterization of renal papillary cells. (A) Immunofluorescence analysis for markers of activated fibroblasts (α-SMA, green; Vimentin, red), mesenchymal stem cells (N-cadherin, green), and epithelial cells (CK18, red; E-cadherin, green; ZO-1, red). Nuclei were counterstained with DAPI (blue) (Magnification: ×100). (B) Immunofluorescence staining for the stem cell expression makers Nestin (green) and CD133 (red). Nuclei are stained with DAPI (blue). (Magnification: ×200). (C) Flow cytometry analysis for the MSC markers CD29 (i), CD90 (ii), and CD73 (iii), and the hematopoietic stem cell marker CD45 (iv).

The cells isolated from the renal papilla exhibited expression of α-SMA and Vimentin (activated fibroblast markers), N-cadherin (mesenchymal cell marker), and Nestin and CD133 (stem cell makers). The epithelial markers expression, including E-cadherin, CK18, and ZO-1, could not be detected (Fig 3A and 3B). Moreover, 99%, 95.8%, and 99.9% of isolated KSCs were positive for the MSC markers CD29, CD90, and CD73, respectively, while only 3.4% were positive for CD45 expression (Fig 3C). This data suggests that the cells isolated from renal papilla may be KSCs.

### Evaluation of the KSC differentiation capacity

**Fig 4:**
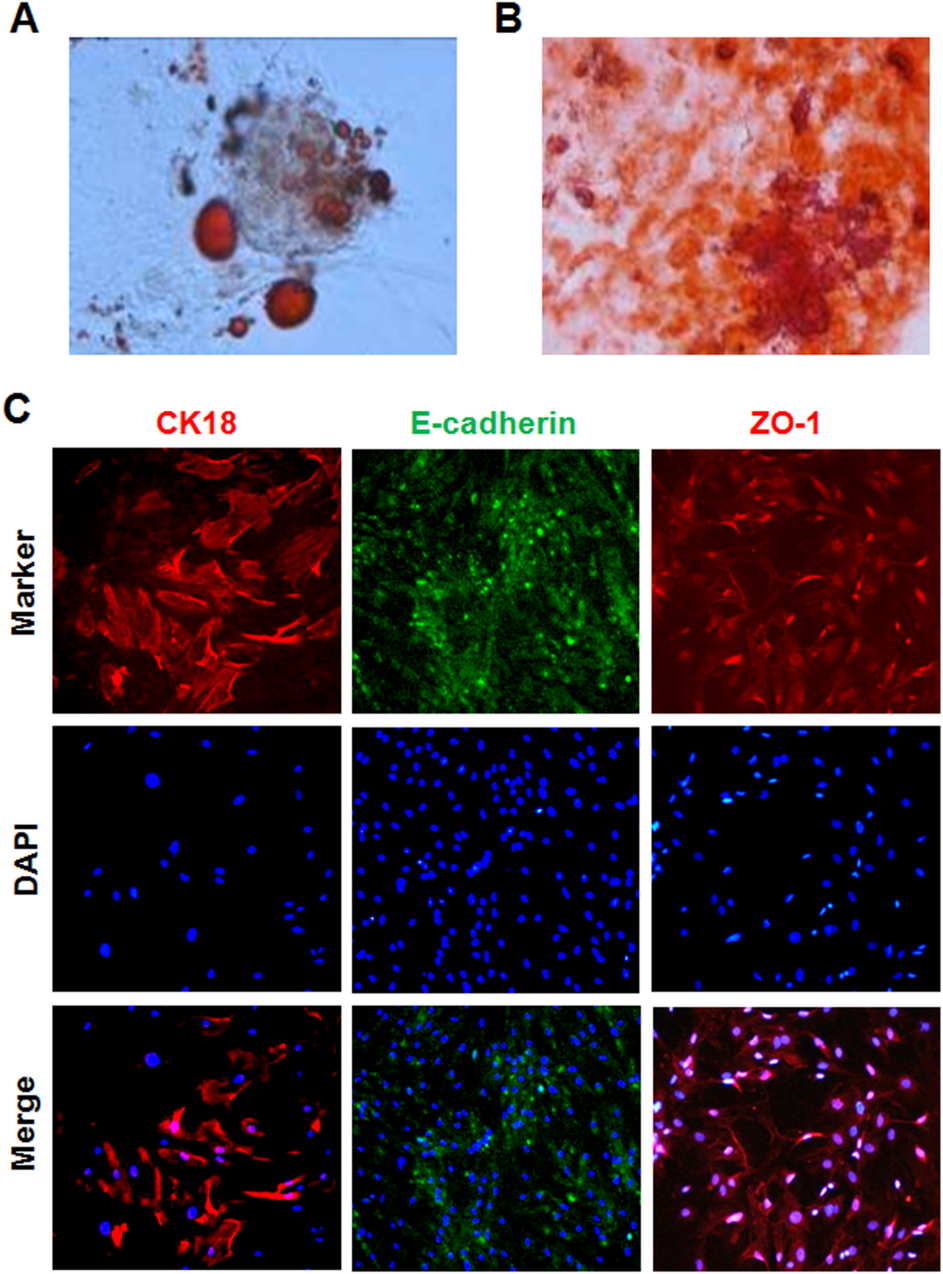
Determination of KSC differentiation abilities. (A,B) KSC adipogenic and osteogenic differentiation was assessed by (A) oil red O and (B) alizarin red staining, respectively (Magnification: ×200). (C) Epithelial cell differentiation was examined by immunofluorescence staining for the mature epithelial cell markers CK18 (red, ×200), ZO-1 (red, ×200), and E-cadherin (green, ×100). Nuclei are counterstained with DAPI (blue).

KSCs subjected to adipogenic differentiation acquired spindle-like morphology, and gradually shrunk into triangular or polygonal shapes. Small lipid droplets formed in cells after seven days of continuous culture induction, which partially merged after two weeks to fill the entire cytoplasm. Induced cells stained positive with oil red O (Fig 4A). Alternatively, KSC culturing in osteogenic medium induced morphological changes into irregular polygons after one week, and small black cytoplasmic particles were observed 2~3 weeks later. Early osteogenesis was demonstrated by positive staining with alizarin red (Fig 4B). Finally, co-culturing with hypoxia-injured RTECs enhanced KSC survival and morphing into round and oval shapes. After 10 days in co-culture, low-level expression of the mature epithelial cell markers CK18, ZO-1, and E-cadherin was observed by immunofluorescence staining (Fig 4C).

### Analysis of embryonic stem cell marker expression in KSCs

**Fig 5:**
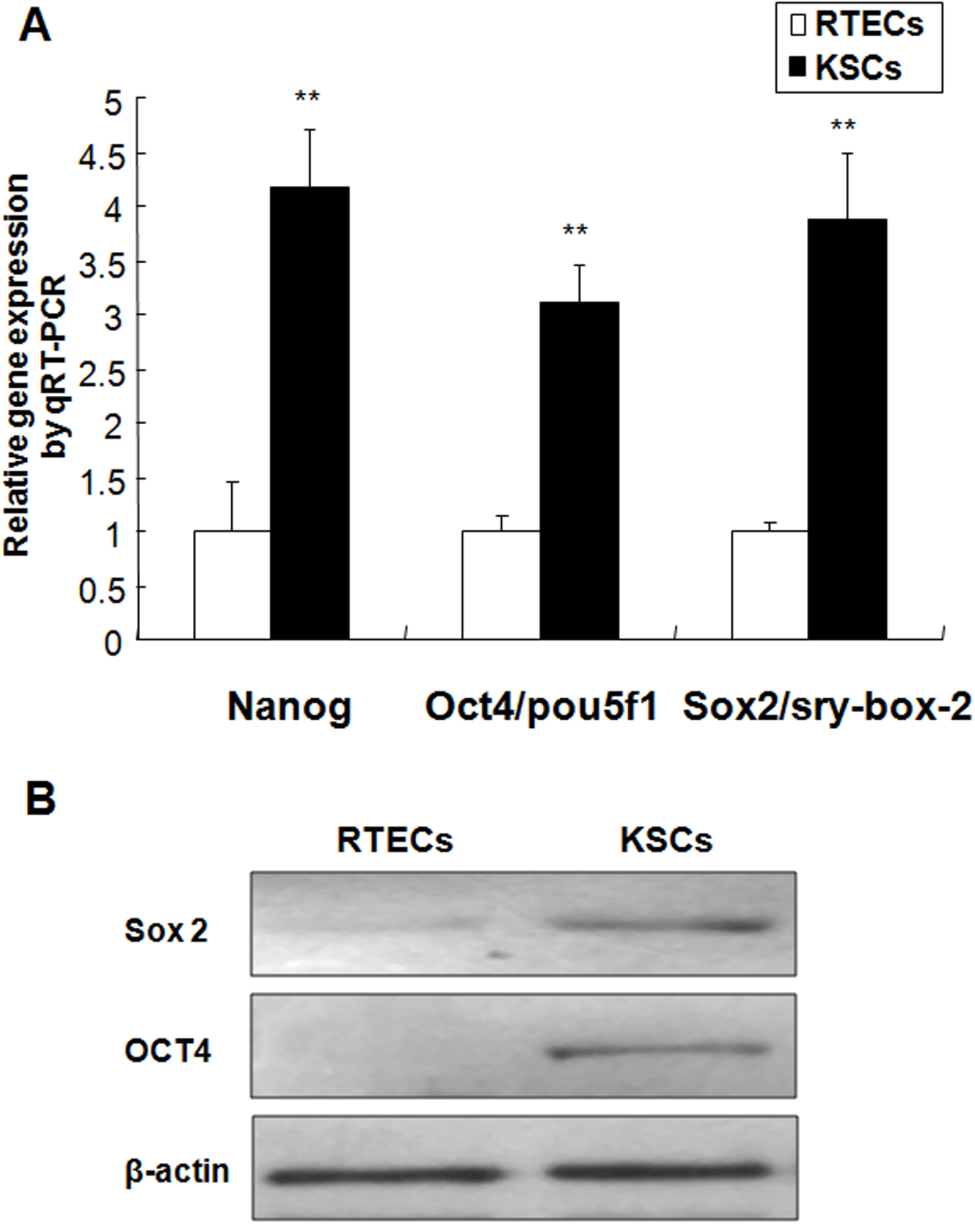
Embryonic stem (ES) cell marker expression in KSCs. (A) *Nanog*, *Oct4/Pou5f1*, and *Sox2* gene expression in KSCs and RTECs. Data represents the mean fold change ± standard error of the mean (SEM) when comparing KSCs to RTECs. All values were normalized to *Gapdh* expression. (**P < 0.01). B: Western blot analysis of ES markers in KSCs and RTECs. β-actin was used as an internal control.

Evaluation of embryonic stem (ES) cell marker gene expression—including that of *Nanog*, *Oct4/Pou5f1*, and *Sox2*—revealed higher levels in the KSCs when compared to RTEC counterparts (Fig 5A). This was consistent with data from Sox2 and Oct4 protein expression analysis (Fig 5B).

### Effects of hyperglycemic stress on KSC proliferation

**Fig 6:**
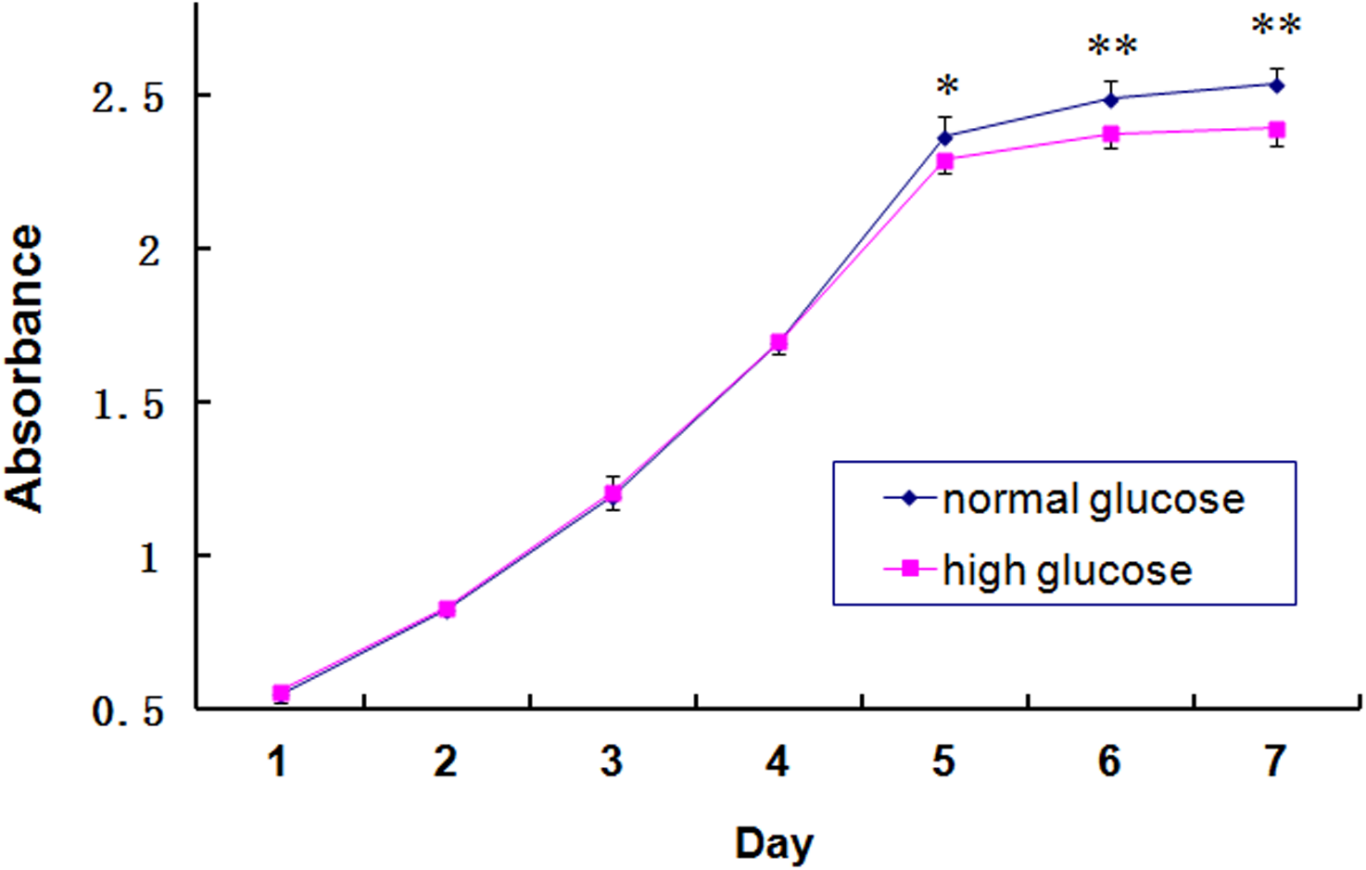
KSC growth curves after culturing in normal-glucose or high-glucose medium. Growth curves for cells cultured in high-glucose or normal-glucose media were graphed based on the OD values at 450 nm. (*P < 0.05, **P < 0.01).

No significant growth differences were observed between KSCs cultured in normal- or high-glucose media—both growth curves appeared similarly S-shaped. Cultures took approximately four days to enter exponential growth and plateaued after 6 days of optimal growth. Specifically, the growth of the high-glucose KSCs was more rapid than that of normal-glucose controls during the first four days, but this difference was not statistically significant. After this point, cell growth in the high-glucose group was significantly slower than that of normal-glucose counterparts (Fig 6). The population doubling times were 58.6 and 56.5 h for the high-glucose and normal-glucose control groups, respectively.

### Influence of hyperglycemic stress on KSC tolerance to hypoxia

The cell cycle analysis of KSCs cultured in high-glucose media revealed a majority of diploid cells, indicative of a low overall viability. After culturing in hypoxic conditions for 24 h, the number of tetraploid cells increased significantly, with a reciprocal decrease in the G0/G1 population. The S phase, G2/M ratio, and overall cell viability increased, suggesting that hypoxia stimulated KSC proliferation. Additionally, lower PI was observed in high-glucose-treated KSCs compared to the normal-glucose-treated control cells (Table 1), suggesting that KSC viability deteriorated in the presence of high glucose.

**Table 1:** Effect of glucose on cell cycle progression.

Group	G0/G1 (%)	S (%)	G2/M (%)	Viability (%)
**NG control**	92.11	5.04	2.85	7.89
**NG hypoxia**	51.54	41.92	6.55	48.46
**HG control**	90.92	3.63	5.45	9.08
**HG hypoxia**	60.26	27.06	12.68	39.74

NG, normal glucose; HG, high glucose.

### Effects of hyperglycemic stress on KSC differentiation into RTECs

**Fig 7:**
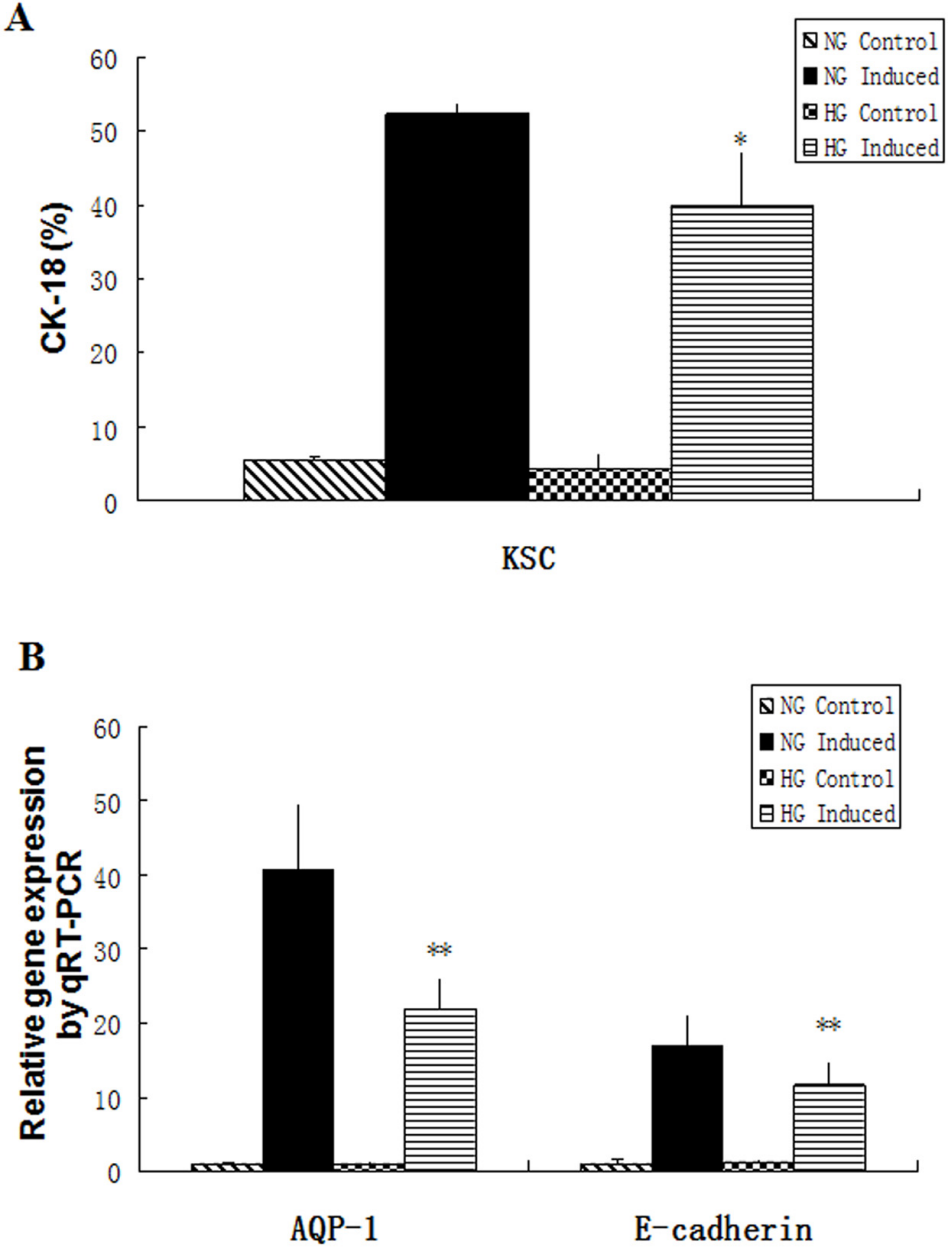
Effects of high glucose on KSC differentiation into epithelial cells. (A) Flow cytometry analysis of CK18 expression (*P* = 0.045); (B) E-cadherin and AQP-1 gene expression analysis by qRT-PCR. (*P < 0.05, **P < 0.01).

Normal- or high-glucose medium-pretreated KSCs were co-cultured with hypoxia-injured RTECs in induction medium to investigate their propensity to undergo epithelial differentiation. Notably, after induction, normal-glucose pretreated KSCs showed a significantly higher expression of CK18 than those of high-glucose pretreated KSCs (52.37 ± 1.23 vs. 39.86 ± 7.44%, *P* < 0.05) (Fig 7A). Moreover, the induction of epithelial E-cadherin and AQP-1 expression in KSCs were markedly increased in co-cultures with injured RTECs when compared to single-culture controls (*P* < 0.01), while compare with high-glucose medium-pretreated KSCs induced group, E-cadherin and AQP-1 expression levels increased more obviously in normal-glucose medium-pretreated KSCs induced group (*P* < 0.01) (Fig 7B).

## Discussion

The macrovascular and/or microvascular complications observed in diabetes involve almost every organ in the body. Stem cells reside in various physiological microenvironment present within the body, and thus are also subjected to the high-glucose environment of diabetes. This results in a decreased stem cell regenerative capacity and an increased susceptibility to AKI. For example, a large-scale epidemiological investigation suggested that the risk of AKI was 2.46 times higher in diabetics than in healthy individuals [3]. Another showed that diabetes was an independent risk factor for defective renal function improvement following AKI [4]. We theorized that the poor kidney reserve capacity identified in diabetes patients resulted from the limited regenerative ability of KSCs; however, a confirmed KSCs population and the effects of a high-glucose microenvironment on KSC function were yet to be determined. In this study, we investigated the phenotypic characteristics of KSCs were investigated in great detail, and confirmed the ability for these cells to differentiate into epithelial cells. Moreover, we also explored the effects of hyperglycemic stress on KSC proliferation, tolerance to hypoxia, and differentiation potential. Significantly, these results showed that the high-glucose microenvironment present in diabetes likely hinders KSC function in many aspects.

To perform these studies, we successfully isolated KSCs from the rat renal papilla and achieved steady adherent growth in a cell culture system. KSCs were primarily spindle- or dendritic-shaped, positive for CD29, CD90, and CD73 MSC marker expression, and successfully differentiated into adipocytes, osteocytes, and early epithelial cells. These findings were consistent with the MSCs evaluation criteria recommended by the 2006 Mesenchymal and Tissue Stem Cell Committee of the International Society for Cellular Therapy [12]. The renal papilla primarily comprises collection ducts, the medullary loop, and vascular and interstitial cells [13]. Early KSC cultures included both epitheloid and fibroblast-like cells that died shortly after isolation, leaving nearly pure cultures of cells with mesenchymal morphology. We determined that this isolated KSC population was devoid of epithelial E-cadherin, CK18, and ZO-1 expression, but positive for the mesenchymal markers α-SMA, Vimentin, and N-cadherin. This result indicated that KSCs exhibited a phenotype similar to the MSC population identified in a previous study [14]. In addition, the isolated KSCs were partially positive for the expression of the ES cell markers Nestin and CD133, as well as Nanog, Oct4/Pou5f1, and Sox2, which are specifically expressed in multipotent embryonic and adult stem cells [15,16]. This expression pattern was the similar that reported by Ward *et al* [17]. In summary, we successfully isolated and cultured cell population with MSC-like properties from the rat renal papilla.

It is suggested that a high-glucose microenvironment affects MSC proliferation [18,19]. Accordingly, our results demonstrated a decline in cell proliferation ability after culturing in high-glucose medium. Some authors report that high glucose could reduce the proliferative and differentiation ability of bone marrow-derived or umbilical cord MSCs, as well as increase cell necrosis induced by ischemia and hypoxia [18–20]. Several studies have shown that renal papillary KSCs can repair injured RTECs and improve kidney function in response to AKI [9,17]. Our previous study demonstrates KSCs protect and participate in the repair of ischemic reperfusion injury of RTECs [11]; however, the effects of a high-glucose microenvironment on this process have not yet been detailed. Thus, we performed hypoxic intervention studies with KSCs cultured in high- and normal-glucose cultured medium. Notably, cell cycle analysis revealed that the proliferative abilities of hypoxic KSCs were lower in high-glucose medium than that in the normal control medium, suggesting that KSCs were more sensitive to hypoxia when subsisting in a high-glucose environment.

Tissue repair by stem cells primarily results from either cellular paracrine effects or the direct migration of cells to damaged sites, where they differentiate to replace the injured cells [21]. The migration and differentiation abilities of renal papillary KSCs have been demonstrated previously. For example, Ward *et al* discovered that CD133/1^+^ stem cells could integrate with co-cultured RTECs and form hollow, spherical spicule shapes, and were observed to participate in the development of renal tubules after injection into the kidneys of mouse embryos [17]. Moreover, Oliver *et al* observed that BrdU-labeled KSCs migrated out of the renal papilla during AKI and participated in the repair of renal tubular epithelial cells [8,9]. Thus, we induced KSC differentiation in vitro to evaluate their capacity to develop into epithelial cells. These results indicated that the mature epithelial cell marker CD18 was expressed by >50% of the cell population after induction [22]. We used a novel method of alternating induction and REGM medium as an induction system for KSCs differentiation. The main components in the induction medium were BMP-7, activin A, and retinoic acid, all of which are key factors for epithelial cell differentiation [23, 24]. REGM medium was used to help preserve the epithelial cell phenotype. To mimic the microenvironment induced by AKI and increase their differentiation efficiency [25], KSCs were co-cultured with hypoxia-injured RTECs. These studies revealed that the differentiation efficiency of KSCs into epithelial cells diminished from 50% to 30% after pretreated with high-glucose media. Consistently, qRT-PCR analysis also showed that mature epithelial cell marker expression was significantly lower in KSCs pretreated with high-glucose conditions, suggesting that this environment hinders KSC epithelial differentiation. Wang *et al* previously observed that high-glucose environments inhibit the osteogenic differentiation of MSCs via cAMP/PKA/ERK pathway activation [26]. In addition, Stolzing *et al* demonstrated that the reduced ability of MSCs to differentiate into fibroblasts in diabetic rats likely associates with glycation end products that mediate cell apoptosis and senescence [27]. Nevertheless, the precise mechanisms by which hyperglycemic stress lowers the epithelial differentiation capacity of KSCs in our study require further investigation.

## Conclusions

In conclusion, the present study successfully demonstrated that KSCs isolated from the rat renal papilla could differentiate into epithelial cells in vitro. In addition, the high-glucose microenvironment can damage KSC stemness, including their proliferation, tolerance to hypoxia, and epithelial differentiation. Notably, these impairments may result in a diminished reparative capacity of KSCs in the injured kidney.

## Supporting Information

S1 Fig Optimization of the KSC epithelial differentiation protocol. Two methods were compared to induce KSC epithelial differentiation: (1) KSCs cultured alone in differentiation medium and (2) KSC/hypoxia-injured RTEC co-cultures in differentiation medium. After induction, cytometric analysis showed that KSCs from single cultures were 37.83 ± 7.53% positive for CK18 expression, whereas co-cultured cells were 50.77 ± 11.03% positive (*P* = 0.022). Thus, the later method was used in the final analyses. (**P < 0.01, #*P* < 0.05).
(TIF)
