# Supplementary figures and images for: Hyperglycemic Stress Impairs the Stemness Capacity of Kidney Stem Cells in Rats
Source: PLoS One. 2015 Oct 2;10(10):e0139607. doi: 10.1371/journal.pone.0139607 (PMC4592017; doi:10.1371/journal.pone.0139607)

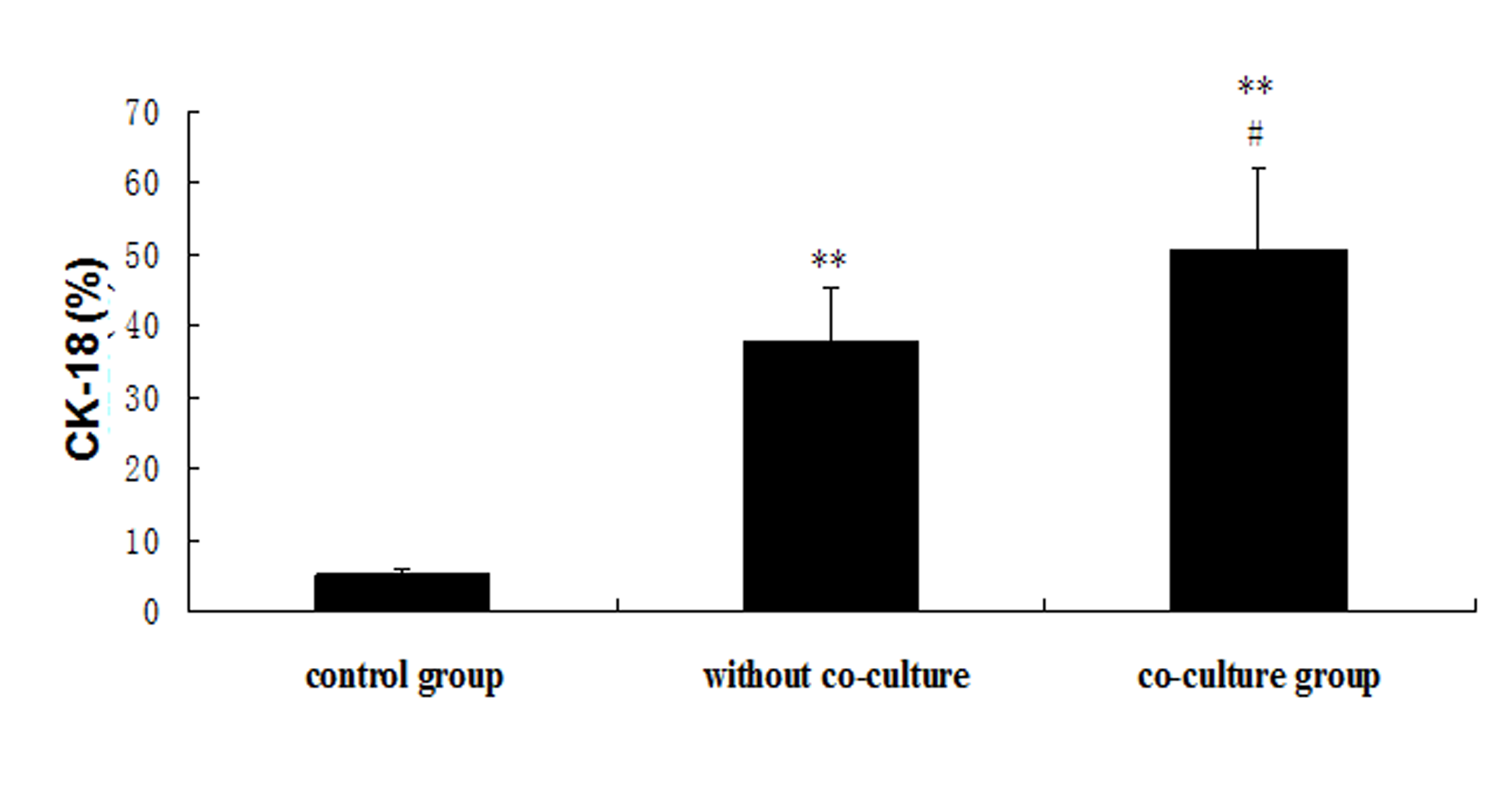

Supplement: S1 Fig — Two methods were compared to induce KSC epithelial differentiation: (1) KSCs cultured alone in differentiation medium and (2) KSC/hypoxia-injured RTEC co-cultures in differentiation medium. After induction, cytometric analysis showed that KSCs from single cultures were 37.83 ± 7.53% positive for CK18 expression, whereas co-cultured cells were 50.77 ± 11.03% positive (P = 0.022). Thus, the later method was used in the final analyses. (**P < 0.01, #P < 0.05). (TIF) [file pone.0139607.s001.tif]
